# Effectiveness of multi-modal cognitive behavioural therapy in improving mental well-being among caregivers of children with disabilities in urban Uganda: A cluster-randomized controlled trial

**DOI:** 10.7189/jogh.12.04102

**Published:** 2022-12-29

**Authors:** Mariam Namasaba, Sumaya Nabunje, Ali Ayub Baguwemu

**Affiliations:** 1 Department of Psychology, Kyambogo University, Kampala, Uganda; 2 Sanlam Life Insurance Limited, Kampala, Uganda

## Abstract

**Background:**

In Sub-Saharan Africa, 41 to 58% of the caregivers of children with disabilities experience psychological distress and have poor mental well-being. Cognitive behavioural therapy (CBT) has a moderate effect on improving mental well-being. However, no study has examined its effects among caregivers of children with disabilities at home and in schools. This study evaluated the effectiveness of CBT in improving mental well-being among caregivers of children with disabilities in urban Uganda.

**Methods:**

We conducted a two-arm cluster-randomized controlled trial in 11 schools across the Kampala district of Uganda. The intervention was a multi-modal CBT training program conducted for six months among 392 home and school caregivers of children with disabilities. In the first three months, caregivers received group-based CBT, and in the next three months, they received phone-based CBT. We used generalized linear mixed-effects regression to examine the differences in the mental well-being of caregivers in the control group vs those in the intervention group.

**Results:**

Home caregivers’ mental well-being was significantly higher after phone-based CBT (unstandardized coefficient of the estimate (B) = 4.31, 95% CI = 1.18-6.82; *P* < 0.001, Cohen’s D (*d*) = 0.27). School caregivers’ mental well-being was significantly higher after group-based CBT (B = 3.98, 95% CI = 0.22-7.47; *P* = 0.038, *d* = 0.25).

**Conclusions:**

Group-based CBT improved mental well-being among school caregivers, and phone-based CBT improved mental well-being among home caregivers. Interventions targeting school caregivers of children with disabilities should employ group settings and those targeting home caregivers should utilize peer-to-peer networks to enhance the caregivers’ mental well-being.

**Registration:**

The study protocol was registered with UMIN Clinical Trials Registry (UMIN-CTR). Trial ID: UMIN000040912.

The mental well-being of caregivers of children with disabilities is a global health concern. Globally, over 38% of caregivers of children with disabilities have clinical depression [1,2], and 41 to 58% in Sub-Saharan Africa face psychological distress [3]. In low- and middle-income countries (LMICs), the caregivers face up to 15% increased financial burden because of their children’s disabilities [4]. The burden of poor mental well-being among caregivers of children with disabilities is amplified in LMICs because of 1) a lack of financial resources, 2) scarcity of trained mental health professionals, and 3) a negative outlook on disability [5,6]. These precarious dynamics cause heightened stress and anxiety for home and school caregivers, resulting in poor mental well-being [5,7].

In Sub-Saharan Africa, only about 10% of the population can access mental health care or evidence-based treatments [3]. The lack of access is partly due to insufficient numbers of trained mental health care professionals. For instance, one occupational therapist is available for every 100 families of children with disabilities in Uganda [8]. Moreover, approximately 88% of the families cannot meet their daily necessities, let alone afford professional health care for their children [8,9]. In addition, many communities in Sub-Saharan Africa still harbour negative attitudes towards disability and consider having a child with a disability a curse [10,11]. In response to these challenges, home caregivers in Sub-Saharan Africa, including Uganda, sometimes send their children to schools because they believe schools are a safe abode for them.

At school, children with disabilities receive care primarily from school caregivers [12,13]. School caregivers are those designated to support children with disabilities at school or teachers of the children [13]. In Uganda, school caregivers often feel ill-equipped to provide adequate care for children with disabilities because most do not receive formal training in special needs education [11,12]. They sometimes face difficulties bonding with children with disabilities due to sudden changes in the children’s behaviour [14,15], face unrealistic expectations, and feel unappreciated by home caregivers [16,17]. Upon the recommendation of the World Health Organization, the government of Uganda adopted community-based rehabilitation (CBR) to overcome these challenges [18]. However, because of financial and infrastructural constraints, few (35% of 2 million) families of children with disabilities in Uganda can access CBR services at local health centres [19,20]. A lack of access to CBR services perpetuates the challenge of poor mental well-being among caregivers. Interventions, such as cognitive behavioural therapy (CBT), could democratize access to mental health care and improve caregivers’ mental well-being and their ability to care for children with disabilities [21-23].

CBT is a treatment package that aims to reduce individuals’ negative beliefs about themselves or their capabilities [24,25]. A meta-analysis of 16 systematic reviews spanning 322 clinical trials found that CBT interventions were moderately superior to anti-depressants for treating depression in adults [26]. Moreover, one study reported that CBT interventions effectively reduced 16 psychological disorders, including generalized anxiety and psychological distress [26]. However, there remains much uncertainty about how the benefits of CBT may vary among different groups, such as home and school caregivers of children with disabilities.

Understanding the effectiveness of different CBT modalities is crucial for researchers and policymakers to plan effective interventions for caregivers of children with disabilities. Previous studies offer no insight into the effectiveness of different modalities of CBT among groups of people who face similar challenges [24-27]. No study has investigated the efficacy of CBT delivered by trained therapists vs peer mentors. There is also no evidence on the effects of non-treatment aspects of CBT, such as group settings vs phone-based settings, on the mental well-being of caregivers of children with disabilities [27]. We hypothesized that group-based CBT, led by trained therapists, and phone-based CBT, led by peer mentors, would have distinct benefits for home and school caregivers. Thus, this study examined the effectiveness of a multi-modal CBT intervention for improving the mental well-being of caregivers of children with disabilities in Kampala district, Uganda.

## METHODS

### Study design and setting

The study was a cluster-randomized controlled trial to examine the effectiveness of multi-modal CBT in improving the mental well-being of caregivers of children with disabilities. We followed a two-group, parallel-arm design with equal allocation and variable cluster sizes [28,29]. The clusters were schools of children with disabilities registered by the Kampala Capital City Authority. At the time of the study, Kampala district had 14 schools for children with disabilities, with a potential population of 1000 home caregivers and 350 school caregivers. Of the 14 schools, two were recruited for a pilot study and the remaining 12 for the main study. Of the 12 schools, six were allocated to the intervention group and six to the control group.

Home and school caregivers in the intervention group joined a six-month multi-modal CBT program. In the first three months (April-June 2021), the caregivers received group-based CBT led by trained therapists. In the next three months (July-September 2021), the caregivers received phone-based CBT led by peer mentors. Caregivers in the control group received treatment as usual.

### Study participants and enrolment

The eligibility criteria of the schools and caregivers are shown in **Table 1**. A sample size of 960 caregivers was estimated in OpenEpi version 3, using an intra-cluster correlation coefficient of 0.03, an effect size of 0.35, a 95% confidence interval (CI), and a power of 80% [30]. An additional 25% was included to account for potential attrition. Home and school caregivers in the participating schools were invited to an information seminar by researchers and school administrators. Caregivers who did not wish to be contacted were requested to inform the school’s administration to remove their contacts from the lists before they were available to researchers. After obtaining the contact lists, all caregivers were invited to an information seminar to join the study.

**Table 1:** Eligibility criteria of the clusters and participants enrolled in the study

Eligibility criteria	Exclusion criteria
**Clusters (schools)**	
Located in the Kampala district	Did not meet any one of the inclusion criteria
Licensed and registered with Uganda National Examinations Board (UNEB) to conduct primary-leaving examinations	
Did not participate in the pilot study	
Admits children with disabilities	
**School caregivers**	
Had a direct caring role for a child with a disability in the invited school	Was not a home caregiver of a child with disabilities
Had at least six months of experience working in the selected school	Did not meet any one of the eligibility criteria
Aged 18 years or older	
Proficient in either Luganda or English	
**Home caregivers**	
Were the primary caregiver of a child with a disability at home	Was not a school caregiver of a child with disabilities
Provided care for a child with a disability for at least six months	Had a child with a disability who was not enrolled in the selected school
Aged 18 years or older	Did not meet any one of the eligibility criteria
Proficient in either Luganda or English	

### Randomization and masking

Schools were stratified based on their type (inclusive or special needs) and potential population size. A computerized program, “Randomization in Treatment Arms,” with a 1:1 allocation ratio was used to assign schools to either the control group (n = 6) or the intervention group (n = 6). Allocation was concealed from schools, trainers, research assistants, and participants until recruitment was completed. Study participants and trainers were not blinded due to the educational nature of the intervention [31]. Caregivers gave their consent to participate in the study before randomization, and they received an intervention based on the allocation of the school.

### Intervention

The intervention was a multi-modal CBT program conducted over six months [32]. The CBT modules were selected from the manual “a therapist’s guide to CBT” [33]. Trained therapists and peer mentors delivered the modules separately in two distinct phases. The structure of the CBT program is shown in **Table 2**.

**Table 2:** Structure of the multi-modal cognitive behavioural therapy (CBT) program

	Session content	Module	Mode of delivery
**Phase one: group-based CBT, led by a trained therapist**			
Session 1	Introduce caregivers to the CBT modules	Conceptualizing existing challenges	Group discussions
		Goal setting	
Session 2	Identify the coping skills used by caregivers	Reframing negative attitudes toward caring for children with disabilities	Role plays to practice positive reframing as a coping skill
		Setting individual and group goals	
Session 3	Identify real-world situations to apply identified solutions	Adapting solutions to context-specific problems (at school and at home)	Group discussions and role plays
Session 4	Assess group concerns	Conceptualizing existing challenges	Small-group discussions with a therapist
		Goal setting	
Sessions 5 and 6	Integrate individuals’ concerns with group goals	Modification of the group modules	Role-plays and group discussions
		Assignment of mentors to small groups	
**Phase two: phone-based CBT, led by peer mentors**			
First month	Training in non-confrontative communication	Providing emotional support to address specific challenges	Phone calls and text messages
	Reporting on mentor-mentee relationship and progress	Group discussion of the play Ensiitaano (chaos)	Individual counselling
Second month	Development of personalized coping plans for school and home challenges	Directive support to create individual plans and setting specific outcomes for all caregivers	Reflective writing or dictation using phone recorders
	Setting individual-specific monthly goals		
Third month	Gaining insight on specific problems and brainstorming solutions as a group	Solution-focused dialogue	Feedback from a peer mentor

CBT – cognitive behavioural therapy

#### Phase one: Group-based cognitive behavioural therapy

Caregivers were divided into groups of 15-20 and received the intervention in their assigned cohorts. One occupational therapist and a clinical psychiatric officer delivered the CBT modules following a standard training manual. Home and school caregivers received bi-monthly sessions for an hour. The caregivers used exercise books and pencils for taking notes or drawing pictures to depict emotions that were difficult to express verbally. They also used the exercise books to complete homework assignments at the end of each group session. The caregivers used both English and Luganda during the group activities. Each group-based CBT session lasted 60-80 minutes.

#### Phase two: Phone-based cognitive behavioural therapy

Peer mentors delivered phone-based CBT by making phone calls at the beginning of each month and sending follow-up messages at the end of the month. The peer mentors used a logbook detailing topics for phone discussions and structured text messages. Peer mentors then made monthly reports that were used for guided group discussions with their mentees.

#### Intervention development

The modules for group-based and phone-based CBT were examined for cultural relevance and acceptability in a pilot study. Four experts, two from Kyambogo University and two from local non-governmental organizations (Cheshire services and L'Arche Uganda), monitored and evaluated the pilot study and recommended substituting activities such as breathing and meditation with singing, dancing, and using local dramas to stimulate discussions among caregivers.

#### Conditions for the control group

Caregivers in the control group received treatment as usual from schools. Typically, schools for children with disabilities in the Kampala district, Uganda, provide directive and non-directive counselling through guidance, emotional support, and sign language classes for home and school caregivers. Counselling is provided only by school nurses who do not use specific psychotherapeutic treatments but can write hospital referrals for caregivers who need mental health care services. At the time of the study, the nurses conducted counselling services on the phone. Phone calls between nurses and caregivers lasted an average of 20 minutes.

### Outcome measures

The primary outcome was mental well-being, measured by the Warwick Edinburgh Mental Well-being Scale (WEMWBS) [34]. We assessed mental well-being scores at the individual level rather than the cluster level. We examined differences in caregivers’ mental well-being at three intervals: baseline, three months follow-up (immediately after phase one; group-based CBT), and six months follow-up (immediately after phase two; phone-based CBT).

Two language experts from Makerere University, Uganda, translated the WEMWBS from English to Luganda (the most spoken language in urban Uganda). A third translator from Naruto university, Japan, back-translated it for confirmation. The forward and back translation processes underwent five iterations through discussions among the three language experts and researchers. The final draft of the translated questionnaire was pre-tested in a pilot study with 20 caregivers from two schools. The Luganda WEMWBS contained 14 items rated on a 4-point Likert scale. Caregivers used a questionnaire either in Luganda or English.

### Socio-demographic characteristics

Caregivers’ socio-demographic characteristics were assessed at baseline. The data were categorized into continuous variables (age and years of education) and categorical variables (sex, religious affiliation, employment, forms of social support, and family type).

### Data collection

Data were collected using a self-reported questionnaire. Three trained research assistants facilitated data collection by reading aloud questions for caregivers who could not read or preferred to have the questions read to them. The research assistants demonstrated how to select responses by circling or ticking. Caregivers used 40-60 minutes to complete the survey. 

### Statistical analysis

Intention-to-treat analyses were conducted separately for school caregivers and home caregivers. Data from 6 clusters in the intervention group and 5 clusters from the control group were used in the analysis.

#### Preliminary analyses

First, we used histograms to gain a high-level view of the distribution of the outcome variable: mental well-being. Then we performed a Shapiro-Wilk test to examine the normality of the residues of the outcome variable [35,36]. We considered data to be normally distributed if the *P*-value was above 0.05 or skewed if the *P*-value was below 0.05. We used the Wilcoxon rank-sum tests with continuity correction to check for differences in median outcomes of caregivers in the intervention group and control group at baseline, three months, and six months.

#### Mixed-effects regression analysis

Variables that were significantly associated with the outcome (based on the Wilcoxon rank-sum tests) were entered into generalized linear mixed-effects regression models to assess the effectiveness of the intervention on caregivers’ mental well-being. All analyses were conducted using RStudio (Packages: Tidyverse, lme4, Lavaan, sensemakr, and simr) at 5% significance level [37]. We considered the results statistically significant when the *P* value was lower than 0.05.

#### Ethical considerations

The study conformed with the principles outlined in the Declaration of Helsinki for clinical trials. Participation was voluntary, and caregivers could withdraw from the study at any time. All caregivers who participated in the study provided written informed consent. Those who could not write used a fingerprint on the consent forms. All data were collected in private classrooms. Research assistants and field coordinators were recruited from Kyambogo University Uganda from the special needs education faculty to promote diversity and inclusion. Moreover, the peer mentors who led phone-based CBT were selected from the study’s participants. The study findings have been shared with all participating schools and the Kampala Capital City Authority.

## RESULTS

### Flow of participants through the study

Of 852 caregivers that were invited, 573 (67.2%) accepted to join the study. Of them, 447 were home caregivers, and 126 were school caregivers. After allocation, one school in the control group (55 caregivers) dropped out, citing administrative reasons. Thus, the study included 11 schools and analysed data from 392 caregivers. Before the intervention, the intra-cluster correlation was 0.067. The baseline characteristics of the clusters (schools) are presented in Table S1 in the **Online Supplementary Document**.

### Baseline characteristics of the participants

**Figure 1:**
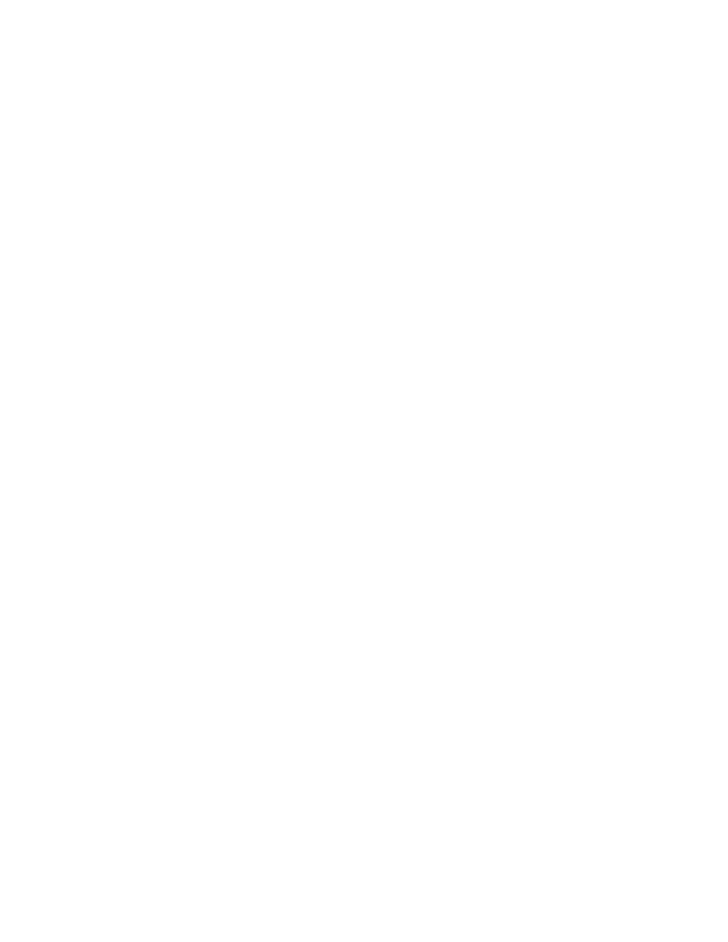
Flow of participants through the main study.

Out of 392 caregivers, 50.7% of the home caregivers and 48.3% of the school caregivers attended all six group-based CBT sessions. Of all, 80% of the caregivers received phone-based CBT. At three months follow-up, 84.7% responded to the survey, while 83.9% responded at six months follow-up. There were no statistically significant differences among caregivers lost to follow-up in the intervention group vs those in the control group. The flow of participants through the study is shown in **Figure 1**.

**Table 3** shows the baseline characteristics of the caregivers. Home caregivers in the intervention and control groups were similar across education levels and religious affiliations. More than 50.0% had vocational education, and approximately one-third (34.0%) were protestant by religion. Home caregivers in the intervention group had a mean age of 42.3 (standard deviation (SD) = 11.4) years, and those in the control group had a mean age of 40.8 (SD = 11.4) years. About one-third (27.9%) of the caregivers in the intervention group were unemployed.

**Table 3:** Baseline characteristics of caregivers by allocation and subgroup (n = 392)

Variables	Description	Control n (%)	Intervention n (%)	Total n (%)
**Home caregivers**				
Age (years), mean (SD)		40.8 (11.4)	42.3 (11.1)*	
Education level	Incomplete primary	37 (13.9)	47 (17.6)	78 (29.3)
	Complete primary	16 (6.0)	27 (10.2)	43 (16.6)
	Incomplete secondary	19 (7.1)	34 (12.8)	53 (19.9)
	Complete secondary	13 (4.9)	16 (6.0)	29 (10.9)
	Vocational education	31 (11.7)	28 (42.4)	59 (54.1)
Religion	Protestant	40 (15.0	53 (19.9)	93 (34.1)
	Catholic	25 (9.4)	40 (15.3)	65 (24.4)
	Muslim	24 (9.0)	38 (14.3)	62 (23.3)
	Pentecostal	26 (9.8)	19 (7.1)	45 (16.9)
Employment status	Employed	78 (29.3)	72 (27.1)*	150 (56.4)
	Unemployed	37 (13.9)	79 (29.7)	116 (43.6)
**School caregivers**				
Age (years), mean (SD)		38.5 (9.9)	**36.6 (10.1)†**	
Education level	Incomplete secondary	7 (5.3)	6 (4.6)	13 (9.9)
	Complete secondary	3 (2.3)	4 (3.0)	7 (5.3)
	Vocational school	12 (9.1)	28 (21.2)	40 (30.3)
	Complete university	29 (21.1)	37 (28.0)	66 (50.0)
Religion	Protestant	27 (20.5)	34 (25.8)	61 (46.2)
	Catholic	14 (10.6)	25 (18.9)	39 (29.6)
	Muslim	2 (1.5)	8 (6.1)	10 (7.6)
	Pentecostal	8 (6.1)	8 (6.1)	16 (12.1)
**Children**				
Age (years), mean (SD)		11.9 (3.5)	**11.5 (3.8)†**	
	6-13 (Ref)	84 (21.4)	156 (39.8)	240 (61.2)
	14-15	43 (10.9)	47 (12.0)	90 (22.9)
	16-18	30(7.6)	32 (00.8)	62 (8.1)
Disability category	Intellectual	40 (10.2)	**64 (16.3)†**	104 (26.5)
	Physical	35 (8.9)	88 (22.4)	123 (31.4)
	Sensory	96 (24.3)	69 (17.6)	165 (42.1)
Disability severity	Mild	5 (1.3)	14 (3.6)	19 (4.9)
	Moderate	69 (17.6)	99 (25.3)	168 (42.9)
	Severe	56 (14.3)	70 (17.9)	126 (32.2)
	Very severe	36 (9.2)	43 (11.0)	79 (20.2)
Children’s gender	Male children	69 (17.6)	96 (24.5)	165 (42.1)
	Female children	97 (24.7)	130 (33.2)	227 (57.9)
**Total n (%)**		**166 (42.3)**	**226 (57.7)**	**392**

SD – standard deviation, Ref – reference group

*P < 0.05.

†*P* < 0.001.

School caregivers in the intervention and control groups were also similar in their levels of education and religious affiliations. The lowest level of education among them was incomplete secondary school (9.9%), and 50.0% of them had completed university. Of all, 46.2% were protestant by religion. School caregivers in the intervention group had a mean age of 36.6 (SD = 10.1) years, and those in the control group had a mean age of 38.5 (SD = 9.9) years.

Caregivers in the intervention and control groups had children similar in gender and severity of the disability. Approximately 60.0% of them had female children, and 43% considered their children’s disabilities moderate. Caregivers in the intervention group had children with a mean age of 11.5 (SD = 3.8) years. Also, 16.8% of the caregivers in the intervention group had children with intellectual disabilities compared to 9.4% in the control group.

### Comparison of the difference in median scores of school caregivers’ outcomes by allocation group and subgroup

**Table 4** shows a comparison of the median differences in caregivers’ mental well-being at three time points. There were no statistically significant differences in home caregivers’ mental well-being at baseline and three months follow-up. However, at six months follow-up, home caregivers in the intervention group had higher mental well-being than those in the control group.

**Table 4:** Between-group comparison of caregivers’ mental well-being over time (mental well-being score range = 14-56)

Outcomes	Control median (IQR)	Intervention median (IQR)	Rank difference (95% CI)	Test-statistic (Wilcoxon)	
**Home caregivers**					
Mental well-being at baseline	46.0 (9.0)	46.0 (9.3)	1. 8 (-1.0 to 2.0)	6622	
Mental well-being at three months	45.0 (10.0)	46.0 (7.0)	-0.1 (-2.0 to 0.1)	5498	
Mental well-being at six months	47.0 (9.0)	48.0 (8.0)	2.0 (4.1-3.1)*	6779	
**School caregivers**					
Mental well-being at baseline	51.0 (6.3)	51.0 (6.0)	-1.2 (-1.1 to 1.0)	1281	
Mental well-being at three months	46.0 (5.0)	47.0 (8.0)	-0.1 (-2.1 to 1.0)*	1419	
Mental well-being at six months	47.0 (6.0)	47.0 (6.0)	0.1 (-1.0 to 2.0)	2064	

IQR – interquartile rage, CI – confidence interval

*P < 0.05.

There were no statistically significant differences in school caregivers’ mental well-being at baseline. At three months follow-up, school caregivers in the intervention group had higher mental well-being than those in the control group. At six months follow-up, no statistically significant differences in school caregivers’ mental well-being were found.

### Effect of the intervention on caregivers’ mental well-being

**Table 5** shows the results of a generalized linear mixed-effect analysis of caregivers’ mental well-being by allocation, time, and subgroup. Immediately after group-based CBT (three months follow-up), home caregivers in the intervention group had higher mental well-being scores than those in the control group, although it was not statistically significant in models 1 and 3. Immediately after phone-based CBT (six months follow-up), home caregivers in the intervention group had higher mental well-being than those in the control group, based on models 1 and 2.

**Table 5:** Generalized linear mixed-effects analysis for the effect of the intervention on caregivers’ mental well-being (mental well-being score range = 14-56)

				Mixed-effects analysis			Effect size
	**Control, mean (SD)**		**Intervention, mean (SD)**	Model 1*, B† (95% CI)	Model 2*, B† (95% CI)	Model 3*, B† (95% CI)	**Cohen’s *d***
**Home caregivers**							
Mental well-being at baseline	46.0 (6.51)	45.7 (6.33)		-	-	-	
Mental well-being at three months	44.5 (6.14)	45.1 (5.77)		0.61 (-0.79 to 2.00)	0.20 (0.12-0.29)	0.13 (0.05-0.2)	0.13
Mental well-being at six months	45.2 (6.78)	47.5 (5.49)		4.31 (1.18-6.82)§	4.34 (1.46-7.40)‡	4.40 (-2.49 to 5.28)	0.27
**School caregivers**							
Mental well-being at baseline	49.9 (4.15)	50.3 (4.11)		-	-	-	
Mental well-being at three months	44.8 (5.09)	45.4 (6.09)		3.98 (0.26-7.70)‡	3.98 (0.26-7.74)‡	2.78 (-1.52 to 5.09)	0.25
Mental well-being at six months	46.6 (5.87)	46.6 (5.72)		1.60 (-1.54 to 4.75)	1.60 (-1.54 to 4.75)	2.29 (0.58-5.16)	0.10

SD – standard deviation, CI – confidence interval

*Model 1: Complete case analysis; fixed effects: age (caregiver and child), disability category, time, allocation, employment status; random effects: caregiver’s identification number (ID). Model 2: Complete case analysis; fixed effects: age (caregiver and child), disability category, time, allocation, employment status; random effects: caregiver’s ID, cluster (school ID). Model 3: Multiple imputation; fixed effects: age (caregiver and child), disability category, time, allocation, employment status; random effects: caregiver’s ID and cluster (school ID).

†Unstandardized coefficient of the estimate.

‡*P* < 0.05.

§*P* < 0.001.

Following group-based CBT (three months follow-up), school caregivers in the intervention group had higher mental well-being than those in the control group, based on models 1 and 2. However, after phone-based CBT (six months follow-up), there were no statistically significant differences in the school caregivers’ mental well-being.

### Factors associated with caregivers’ mental well-being

The study also performed exploratory analyses to determine the associations between caregivers’ baseline characteristics and their mental well-being.

The factors associated with home caregivers’ mental well-being are presented in Table S2 in the **Online Supplementary Document**. Home caregivers of children with sensory disabilities had higher mental well-being than those of children with physical or intellectual disabilities. In addition, home caregivers of older children had higher mental well-being than those of younger children.

The associations between school caregivers’ baseline characteristics and their mental well-being are presented in Table S3 in the **Online Supplementary Document**. There were no statistically significant associations among school caregivers’ characteristics and their mental well-being.

## DISCUSSION

The study examined the effectiveness of multi-modal CBT in improving the mental well-being of caregivers of children with disabilities in urban Uganda. School caregivers’ mental well-being was higher after group-based CBT and home caregivers’ mental well-being was higher after phone-based CBT. Thus, the study confirms the hypothesis that CBT has distinct benefits for home and school caregivers of children with disabilities, based on its mode of delivery.

Group-based CBT effectively improved school caregivers’ mental well-being. However, there was no significant change in the caregivers’ mental well-being after phone-based CBT. Like this study, a previous study reported that group-based CBT was effective for managing work-related stress among adults with depression [38]. The school caregivers in the current study may have been more receptive to group-based CBT than phone-based CBT because they faced similar challenges and could quickly build a shared identity and social cohesiveness [39]. In group-based CBT, social cohesiveness acts as a springboard that launches participants toward improved mental well-being [27]. Therefore, the individualized nature of phone-based CBT was not a good crucible for managing the work-related stress that school caregivers of children with disabilities commonly face.

Home caregivers’ mental well-being was higher after phone-based CBT but not group-based CBT. This finding contrasts with a previous study among caregivers of people with dementia and depression, where the effects of group-based CBT gradually improved but not for phone-based CBT [40]. The contrast is likely because in this study, phone-based CBT was led by peer mentors who were also caregivers of children with disabilities. Given that caregivers of children with disabilities face varied challenges, the peer mentors in this study were well-equipped to provide targeted psychosocial support, improving their mentees’ well-being [41]. It follows that group-based CBT may not have been sensitive to the vast challenges associated with caring for children with varied disabilities, as was the case for home caregivers in this study [27]. The influence of variation in home caregivers’ challenges was reflected in the differences in their mental well-being, based on the type of disability of their children.

Unlike previous studies, this study combined four CBT techniques of cognitive reframing, problem-solving, relaxation, and behavioural activation. Earlier studies focused on either one or a combination of the four techniques [42,43]. This study shows that incorporating all four CBT techniques in an intervention improves mental well-being of caregivers of children with disabilities at home and school. Thus, the study highlights the possibility of expanding CBT’s current application beyond single techniques and delivery by trained therapists. Moreover, the study’s approach increases the potential to reach more people needing mental health care services, including caregivers of children with disabilities.

### Strengths and limitations

The study’s findings have been interpreted considering the following biases. First, the study could not achieve the targeted sample size of 960 participants. The low recruitment rate was caused by the prolonged closure of schools and a total ban on internal travel in Uganda during the study period. Second, the study employed self-reported measures. Respondents may have exaggerated or underreported their responses, affecting the validity of estimates [44] However, the translated WEMWBS used in the study showed good internal validity. Therefore, this study has availed tools that other researchers can use in Uganda. Third, the study was conducted for a short time. Given that the intervention and time interaction was positive and statistically significant for home caregivers’ mental well-being, it is possible that the actual effect of the intervention is greater than what was observed in this study [45] Future studies could conduct the intervention for a longer time span to determine its effects over time. Fourth, the study included only caregivers of children in schools. These caregivers may be different from those whose children are not enrolled in school and therefore experience different effects of the intervention. Future studies can now explore the effects of multi-modal CBT among caregivers of children with disabilities in school and in the community. Lastly, the absence of blinding of participants and therapists may have caused contamination between caregivers in the intervention group and those in the control group [32]. Such contamination lowers a study’s ability to conclude on the effectiveness of a trial [46]. However, contamination reflects the reality of caregivers and indicates that the intervention is relevant and acceptable for them. 

## CONCLUSIONS

Group-based CBT was effective in improving the mental well-being of school caregivers, and phone-based CBT was effective in improving the mental well-being of home caregivers of children with disabilities. CBT interventions for school caregivers should employ group settings and those for home caregivers should utilize peer-to-peer networks to improve the caregivers’ mental well-being. Mental health practitioners, researchers, and policymakers should apply the study’s approach to enhance access to mental health care services for marginalized populations in low-income countries, including caregivers of children with disabilities in Uganda.

## Additional material


Online Supplementary Document
