# Supplementary material for: Effectiveness of multi-modal cognitive behavioural therapy in improving mental well-being among caregivers of children with disabilities in urban Uganda: A cluster-randomized controlled trial
Source: J Glob Health. 2022 Dec 29;12:04102. doi: 10.7189/jogh.12.04102 (PMC9798245; doi:10.7189/jogh.12.04102)
Supplement: Online Supplementary Document [file jogh-12-04102-s001.pdf]

Preliminary results

Internal consistency validity of the questionnaire

The *Luganda* Warwick Edinburgh Mental Well-being Scale (WEMWBS) had strong internal reliability (Cronbach's alpha of 0.813; 95% CI = 0.77 to 0.85, n = 344).

Table S1: Baseline characteristics of the clusters (schools)

Variables	Description	Control<br>n (%)	Intervention<br>n (%)	Total<br>n (%)
School type				
	Inclusive	4(36.4)	4(36.4)	8(0.8)
	Special needs	1(9.1)	2(18.2)	3(1.0)
School setting				
	Boarding school	1(9.1)	1(9.1)	2(18.2)
	Day school<br>(Daily commute by students)	4(36.4)	5(45.5)	9(81.2)

Table S2: Factors associated with home caregivers' mental well-being (n = 266)

Time	Variable	B	Standard error	Statistic (t)
**Model 1**	Caregiver age	-0.022	0.029	-0.550
	Age child	**0.353***	**0.136**	**2.606**
	Unemployed	0.433	0.886	0.489
	Intellectual disabilities	1.577	3.427	0.460
	Physical disabilities	1.125	0.888	1.266
	Sensory disabilities	**3.434****	0.901	3.811
3 months follow-up	Intervention*Time	-3.144	0.732	-4.291
6 months follow-up	Intervention*Time	-0.494	0.942	-0.523
**Model 2**				
	Caregiver age	-0.024	0.028	-0.867
	Age child	0.133	0.081	1.646
	Unemployed	-7.718	1.983	-3.890
	Intellectual disabilities	0.964	3.287	0.293
	Physical disabilities	0.490	0.890	0.551
	Sensory disabilities	**2.136***	1.017	**2.100**
3 months follow-up	Intervention*Time	0.865	0.702	1.232
6 months follow-up.	**Intervention*Time**	**3.192***	**0.731**	**-4.363**
**Model 3**				
	Caregiver age	-0.001	0.029	-0.229
	Age child	0.105	0.089	1.180
	Unemployed	-0.348	0.660	-0.522
	Sensory disabilities	1.483	1.174	1.259
	Physical disabilities	0.498	0.999	0.499
3 months follow-up	Intervention*Time	0.422	0.667	0.633
6 months follow-up.	Intervention*Time	-0.968	0.721	-1.345

B- unstandardized co-efficient; SD-standard deviation; *: *P*-value < 0.05, **: *P*-value < 0.001

Table S3: Factors associated with school caregivers' mental wellbeing

Time	Variable	Estimate (B)	Standard error	Statistic (t)
**Model 1**	Caregiver age	-0.040	0.045	-0.885
	Age child	0.352	0.147	2.396
	Physical disabilities	-0.312	1.069	-0.292
	Sensory disabilities	-0.863	0.880	-0.980
3 months follow-up	Intervention*Time	-1.990	1.110	-1.792
6 months follow-up	Intervention*Time	-0.853	0.910	-0.928
**Model 2**	Caregiver age	-0.040	0.045	-0.885
	Age child	0.352	0.147	2.396
	Physical disabilities	-0.312	1.069	-0.292
	Sensory disabilities	-0.863	1.069	-0.980
3 months follow-up	Intervention*Time	-1.990	1.110	-1.792
6 months follow-up	Intervention*Time	-0.853	0.919	-0.928
**Model 3**	Caregiver age	-0.035	0.036	-0.963
	Age child	0.004	0.120	0.034
	Physical disabilities	-0.210	0.309	-0.550
	Sensory disabilities	-0.492	0.941	-0.523
	(Ref: Intellectual disabilities)			
3 months follow-up	Intervention*Time	-0.930	1.010	-0.921
6 months follow-up	Intervention*Time	1.436	0.750	1.917

B- unstandardized co-efficient; SD- standard deviation; *: *P*-value <0.05, **: *P*-value < 0.001
